# Widowhood and Mortality: A Meta-Analysis

**DOI:** 10.1371/journal.pone.0023465

**Published:** 2011-08-17

**Authors:** J. Robin Moon, Naoki Kondo, M. Maria Glymour, S. V. Subramanian

**Affiliations:** 1 Department of Society, Human Development, and Health, Harvard School of Public Health, Boston, Massachusetts, United States of America; 2 Department of Health Sciences, Interdisciplinary Graduate School of Medicine and Engineering, University of Yamanashi, Chuo-Shi, Yamanashi, Japan; 3 Department of Society, Human Development, and Health, Harvard School of Public Health, Boston, Massachusetts, United States of America; 4 Department of Society, Human Development, and Health, Harvard School of Public Health, Boston, Massachusetts, United States of America; Alberta Research Centre for Health Evidence, - University of Alberta, Canada

## Abstract

**Background:**

While the "widowhood effect" is well known, there is substantial heterogeneity in the magnitude of effects reported in different studies. We conducted a meta-analysis of widowhood and mortality, focusing on longitudinal studies with follow-up from the time of bereavement.

**Methods and Findings:**

A random-effects meta-analysis was conducted to calculate the overall relative risk (RR) for subsequent mortality among 2,263,888 subjects from 15 prospective cohort studies. We found a statistically significant positive association between widowhood and mortality, but the widowhood effect was stronger in the period earlier than six months since bereavement (overall RR = 1.41, 95% CI: 1.26, 1.57) compared to the effect after six months (overall RR = 1.14, 95% CI: 1.10, 1.18). Meta-regression showed that the widowhood effect was not different for those aged younger than 65 years compared to those older than 65 (P = 0.25). There was, however, a difference in the magnitude of the widowhood effect by gender; for women the RR was not statistically significantly different from the null (overall RR = 1.04, 95% CI: 1.00, 1.08), while it was for men (overall RR = 1.23, 95% CI: 1.18, 1.28).

**Conclusions:**

The results suggest that further studies should focus more on the mechanisms that generate this association especially among men.

## Introduction

The death of a spouse is a common experience in old age, and predicts immediate elevation of mortality risk for the surviving spouse [1]. The “widowhood effect,” describing the increased probability of death among those experiencing recent spousal bereavement, is well known. The widowhood effect has been found in bereaved men and women of all ages around the world, particularly in the western industrialized world, using both cross-sectional and longitudinal data, with and without controlling for covariates, and using diverse statistical methodologies [2], [3], [4], [5], [6]. Longitudinal studies put the long-term excess risk of death associated with widowhood compared to marriage at about 15%, net of controls, while estimates of short-term effects during the first few months' immediately post-bereavement range from 50% to 90% [7], [8], [9]. However, there is substantial heterogeneity in the magnitude of effects reported in different studies [7], [9]. A recent meta-analysis of the literature on marital status (including widowhood) and mortality, reported that widowed persons had an 11% higher risk of mortality when compared to married persons after adjustment for age and additional covariates. This meta-analysis compared mortality among elderly widowed and non-widowed and thus left two important questions unanswered. First, does the relationship hold in younger populations? Second, what is the magnitude of the relationship when analyses focus on prospective studies with follow-up from the time of bereavement? Because of the evidence that the risk associated with widowhood changes as time since bereavement elapses, comparing mortality experience of widowers to non-widowers can give very biased estimates of the effect of widowhood. In any existing population, the majority of widowers have already survived the highest risk period, which occurs immediately post-bereavement. It is therefore important to focus on studies that begin follow-up at the time of bereavement, rather than comparing the mortality experience of widowers to non-widowers. We therefore conducted a meta-analysis of 2,263,888 subjects based on similar study selection criteria as the review by Stroebe and colleagues [10], but focusing on longitudinal studies with follow-up from the time of bereavement and including publications since early 2006. We aimed to assess the overall relationship between bereavement and mortality based only on longitudinal studies and also explore the factors explaining heterogeneity among studies published so far. Understanding the sources of such between-study heterogeneity in the existing results is important for interpreting meta-analysis results.

## Methods

### Study Selection

We followed published guidelines for meta-analyses of observational studies [11]. The literature review was conducted in published scientific work to look at the relation between bereavement and mortality. The most valid and reliable information is provided in longitudinal investigations comparing newly bereaved with non-bereaved counterparts, controlling for several confounders such as socioeconomic and lifestyle/behavioral factors the bereaved spouse would have shared with their deceased partner, which could affect the bereaved spouse's health as well.

**Figure 1:**
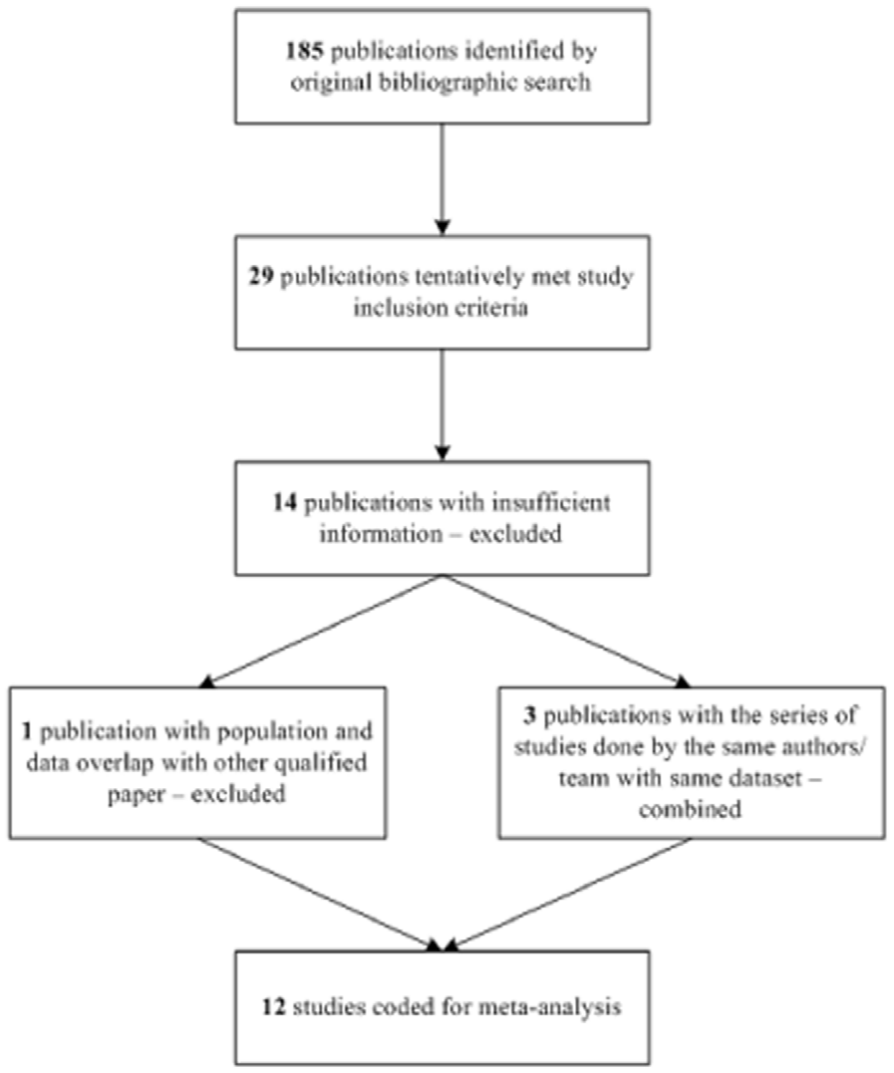
Flow Chart of Study Selection Process.

Studies evaluating widowhood effect and mortality were initially searched using online search engines such as PubMed, Medline, PsycINFO and ISI Web of Science (Thomson Reuters) with the terms “bereavement/grief/grieving/mourn/mourning,” “widow/widows/widowed/ widowhood,” “spouse/spousal” and “mortality/death/survival/longevity” for reports published since 1960 (last searched October 2009; see **Table S1**). During the time period prior to 1990, we found that there were few longitudinal studies. From the studies within this search period, a study was included if it 1) investigated the relationship between mortality and widowhood status; 2) used a population-based sample, i.e., the majority of participants were non-institutionalized and non-hospitalized healthy individuals; 3) reported quantitative data adjusted for or stratified by at least age and gender; 4) was a prospective, longitudinal study beginning mortality follow-up for the widowed group at the time of bereavement; 5) met quality criteria including follow-up time of at least 3 years, response rate of at least 75%, use of standardized measurements, reporting sufficient data to calculate common effect estimates, and use of a control group of non-bereaved individuals; and 6) was published in English. Finally, to avoid duplication of data from the same subjects, information were selected from only one report/analysis of the same dataset. **Figure 1** summarizes the process of selecting studies for the meta-analysis; see **Table S2** for the list of extracted studies.

### Data Extraction

Data from each included article was independently evaluated and extracted by two reviewers (RM and NK), including information on study design, data sources, country of data origin, sample size, the number of cases, age, gender, estimations, response rate, follow-up duration, outcome, adjustment variables, and statistical modeling strategies. In addition, all data needed for the quality assessment and statistical analysis, including relative risk (RR) estimates (in terms of odds ratio, OR; hazard ratio, HR; risk ratio or incidence rate ratio, IRR, and standardized mortality rate, SMR), relative 95% confidence intervals (95% CI) or standard error (SE) for each exposed group were extracted from published reports. Data were also extracted for the sub-studies generated from each main study, and the analysis was stratified by gender and time since the spousal death.

When enrollment spanned over several years, we used the midpoint of the range for analyses that stratified on the study year. In order to analyze whether the effects of bereavement differed by age, we dichotomized age at 65 because this was available for the majority of the studies, i.e., ≥65 = yes = 1; <65 = 0. However, two studies had age dichotomization at 69 [12] or at 74 [13], and we used those ages instead.

### Statistical Analysis

A meta-analysis is an analysis of analyses; it is a statistical method of combining the results of independent studies that address a set of related research hypotheses, exploring heterogeneity, and synthesizing summaries if appropriate, generally aiming at producing a single effect estimate for the purpose of integrating findings [14]. The main objectives of a meta-analysis are to 1) to derive a meaningful overall estimate of effect by increasing power and thus reducing the possibility of concluding that there is no statistical association between widowhood and mortality when in fact there is such an association (type 2 error), and to 2) explore potential sources of heterogeneity of results in existing studies and to discover potential patterns of risk among our study results [15]. Resulting overall averages when controlling for study characteristics can be considered meta-effect sizes, which are more powerful estimates of the true effect size than those derived in a single study under a given single set of assumptions and conditions. Although a relationship between widowhood and mortality has long been recognized, only one prior study [16] has provided a summary estimate of the strength of the association.

As a primary analysis, we estimated the overall relative risk for subsequent mortality among all cohort studies (cohort RR), adjusting for age and gender only. All of the studies we used for our investigation were different in their choices of stratification strategy, whether by age groups (and which age groups), gender, socioeconomic status, or time since bereavement. Not all of them had their “primary” analysis using the overall population controlled for age and gender only. To be more specific, there were 2 studies that showed RR for overall population (male and female combined) for all age groups (Hart, Kaprio); 2 studies with overall population for all age groups stratified by time since bereavement (Hart, Schaefer); 3 studies with gender-stratified population for all age groups (Martikainen, Stimpson, Manor); 3 studies with gender- stratified population by time since bereavement (Martikainen, Kaprio, Nagata); 3 studies with gender-stratified population by age groups (Martikainen, Mineau, Smith); 1 study with gender-stratified population by age groups and socioeconomic status (Martikainen); 1 study with gender-stratified population of all age groups by other charactistics (Christakis); 3 studies with gender-stratified population of all age groups by time-since –bereavement (Christakis, Nagata, Schaefer); 4 studies with gender-stratified population by age groups and time since bereavement (Kaprio, Lichtenstein, Manor, De Leon); and 2 studies with gender-stratified population by age group, time since bereavement and other characteristics (Lichtenstein, Manor). From the RRs adjusted by age and gender only, whether from overall analysis or stratified analysis, we chose from each study a unique set of RRs with the greatest number of deaths, without overlapping the population in order to avoid double-counting. Our primary analysis was conducted using the set of RRs chosen this way. We used the available information from the studies to calculate and convert various effect measures (OR, RR, SMR) to a common effect measure. In other words, we combined the RRs that are already adjusted for or stratified by age and gender, by using the adjusted or age- and/or gender-specific RRs and then combining them using meta-analytic technique. We then estimated the RR for the main effect study from each analysis used. In secondary analyses, we estimated the RRs for subsequent mortality among all cohort studies in the same way, which included additional covariate adjustment. The exact covariates used varied across studies, but included: financial strain, race/ethnicity, US-born, health behaviors (smoking, drinking), FEV1, cholesterol, body mass index (BMI), co-morbidities (diabetes, angina, ischemia, previous myocardial infarction (MI), other cardiovascular diseases, respiratory diseases and other chronic diseases), social class, education, occupation, deprivation categories, relative ages of each spouse, and the number of children. For subgroup analyses, we selected specific models reporting RRs for men or women only, age <65 or ≥65 years only, and time since bereavement <6 months or ≥6 months only, and separately conducted meta-analyses. We evaluated between-study heterogeneity using I^2^ statistics which provides a measure of the percentage of total variability due to between-study heterogeneity, and Cochran Q test which is an extension to chi-square tests for related samples that provides a method for testing for differences between three or more matched sets of frequencies or proportions) [17], [18]. We conducted a sensitivity analysis of whether our primary results were dependent on any single study by repeating analyses excluding each study one at a time. We also conducted a meta-regression, by regressing study-specific effect estimates chosen for our primary analysis on study characteristics as explanatory variables. It should be noted that we used a different set of RRs to conduct the meta-regression on time-since-bereavement variable. These RRs were again a unique set of effect estimates; however, for the purpose of focused analysis on time since bereavement, we chose those with the time-since-bereavement information from each study that had the information available without the regard to the number of deaths. We did this because the set of estimates used in our primary analysis did not include a sufficient number of stratified estimates by <6 months/≥6 months. Finally, we used funnel plots to assess reporting bias, based on the effect estimates used in our primary analysis (stratified single-study results). Begg's and Egger's tests were conducted to measure the degree of funnel plot asymmetry [19], [20]. One of the weaknesses of meta-analysis is the heavy reliance on published studies, which may create exaggerated outcomes, as it is very hard to publish studies that show no significant results. Such a reporting bias results in the distribution of effect sizes that are biased, skewed or cut off, creating a serious base rate fallacy, in which the significance of the published studies is over-estimated.

The meta-analyses were performed in STATA v.11.1 (STATA Corporation, College Station, TX, 2003), including the creation of plots and estimate SE from 95% CI.

## Results

**Table S3** presents an overview of findings of 15 longitudinal studies published since 1960 on the mortality associated with bereavement. The 15 studies got consolidated into 12 studies, because we consolidated 1) three studies by same authors (Martikainen, et al) using the same dataset and 2) two studies by same authors (Christakis & Elwert). The main meta-analysis compared the risk of all-cause mortality of currently married (or persons with a partner) vs. widowed individuals. There was only one study examining the main effect in men and women combined. All other studies gave the results stratified by gender, to show the modifying impact of gender on the outcome. The data were also stratified by age group (dichotomized at 65 years of age), country, and time since bereavement. The studies included in the meta-analysis have the following characteristics. Datasets for the studies come from the USA (7) [7], [8], [13], [21], [22], [23], [24], Finland (4) [9], [25], [26], [27], UK/Scotland (1) [28], Sweden (1) [12], Japan (1) [29], and Israel (1) [30]. Of the 15 studies we used (which eventually became consolidated into 12 distinct studies), thirteen were reported as nationally representative. The number of years of follow-up ranged from 3 to 95 years. The causes of death investigated were mostly all-cause mortality, with 3 studies looking at cardiovascular and cerebrovascular diseases. Most of the studies showed significant excess mortality associated with widowhood, especially in the first month to 6 months following the spousal death. Excess mortality during this period spanned around 40% to 50% on average, for both men and women. Not all studies provided the age- and gender-adjusted data; 8 studies out of 15 only provided the age- and gender-stratified results. Of the 15 studies, 13 papers have some levels of individual socio-economic status (SES) control, using occupation, education, gross income, poverty status and/or housing tenure and condition. Even though SES controls were used for these studies, the level of control was rather coarse, often using dichotomous measures (*e.g.*, below or above the federal poverty line, crude educational categories, etc.). Co-morbidities were controlled in 8 studies, including one study that controlled only the psychiatric disorder prior to the partner's death and only two studies using the Charlson score, the more comprehensive co-morbidities score.

**Figure 2:**
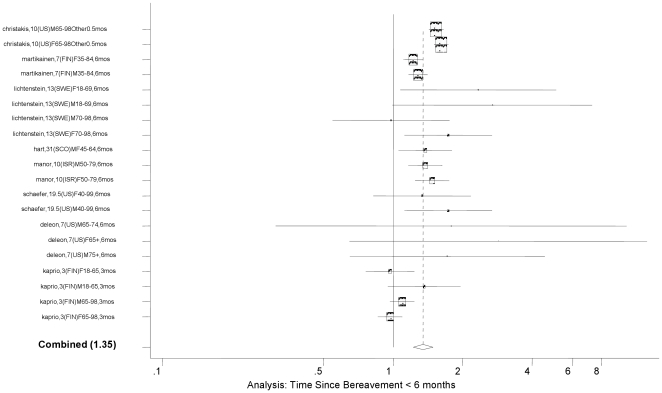
Forest Plot for Time Since Bereavement of <6 months.

The random effect meta-analysis of all eligible age- and gender-adjusted estimates provided an overall RR of bereavement of 1.12 (95% CI: 1.10, 1.15; *P*<0.0001). This result is consistent with the previous meta-analysis result. The studies included in the meta-analysis were highly heterogeneous (I^2^  =  88; 95% CI: 85, 90; Heterogeneity *P*<0.0001). Gender-specific analysis shows that males have overall RR of 1.22 (95% CI: 1.18, 1.26), and females 1.03 (95% CI: 1.00, 1.07). Age-specific analysis shows that those under age 65 years had an overall RR of 1.18 (95% CI: 1.17, 1.25), while those 65 years and above had an overall RR of 1.10 (95% CI: 1.07, 1.13). The overall RR did not change when we selected only those studies that adjusted for additional covariates, e.g., income, education, race/ethnicity, US-born, health behaviors, co-morbidities, social class, occupation, number of children, etc. (overall RR 1.14; 95% CI: 1.11, 1.18; *P*<0.0001; **Table 1**). This was confirmed by meta-regressions that stratified the primary meta-analysis by various covariates. The test of no difference between eight age- and gender-adjusted models and the four models additionally adjusted for other covariates was not statistically significant (*P* = 0.74) (See **Table 2**). As the source of between-study heterogeneity, we found that the widowhood effect was stronger in the period earlier than six months since bereavement (overall RR = 1.41, 95% CI: 1.26, 1.57) compared to the effect in later time (overall RR = 1.14, 95% CI: 1.10, 1.18; *P*<0.0001 for the difference) (See **Figures 2** **and** **3**). In addition, the widowhood effect was not different for those aged younger than 65 years compared to those older than 65 (*P* = 0.25). There was, however, a difference in the magnitude of the widowhood effect by gender; the widowhood effect for women was not statistically significantly different from the null (overall RR = 1.04, 95% CI: 1.00, 1.08, see **Figures 4** **and** **5**), while it was statistically significant for men (overall RR = 1.23, 95% CI: 1.18, 1.28; *P*<0.0001 for the difference). The baseline years of our studies varied from 1860 to 1993. To understand whether there is a cohort effect, we examined the difference in RR depending on the baseline year. There was a small but significant incremental increase in the widowhood effect with calendar year (overall RR = 1.05, 95% CI: 1.01, 1.09). Finally, between-country difference for US vs. non-US was not significant, nor was for US vs. Europe vs. Japan (see **Table 2**).

A series of sensitivity analyses was conducted by omitting one study at a time from the selection pool. The results showed that there was not a particularly influential study among all selected studies (see **Figure 6**). The overall RRs ranged from 1.12 to 1.22. The funnel plot constructed to gauge reporting bias was fairly balanced and the formal statistical test also did not detect any significant reporting bias (Egger's test *P* = 0.35, Begg's test *P* = 0.64). Although producing a funnel plot based on subgroups and not on studies may obscure reporting bias by just including a lot of small non-significant subgroups, in many cases the authors of the selected studies only calculate RRs in subgroups. In this case, the “subgroup analysis” estimates are the appropriate data points for the funnel plot. Further, we had no way of using overall effect size in the funnel plot if the paper does not provide such information.

**Figure 3:**
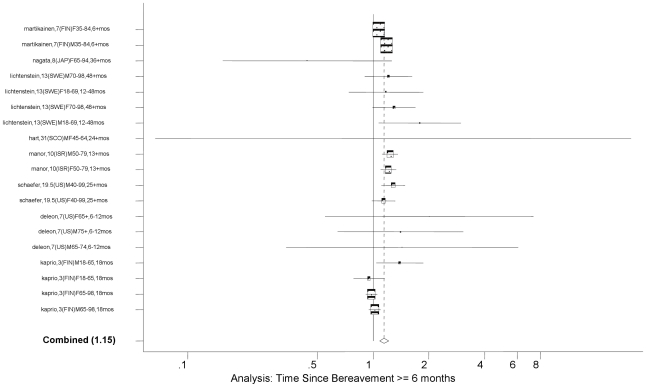
Forest Plot for Time Since Bereavement of ≥6 months.

**Figure 4:**
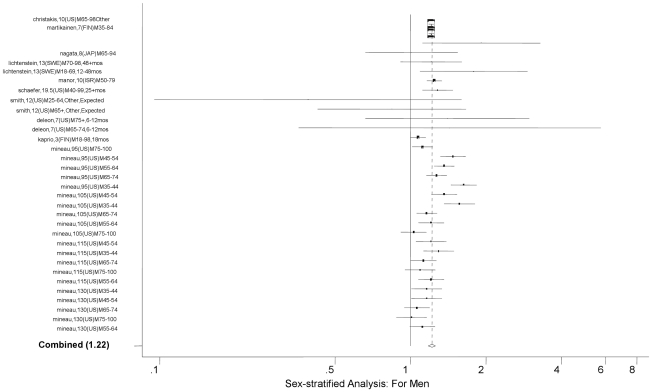
Forest Plot for Men.

**Figure 5:**
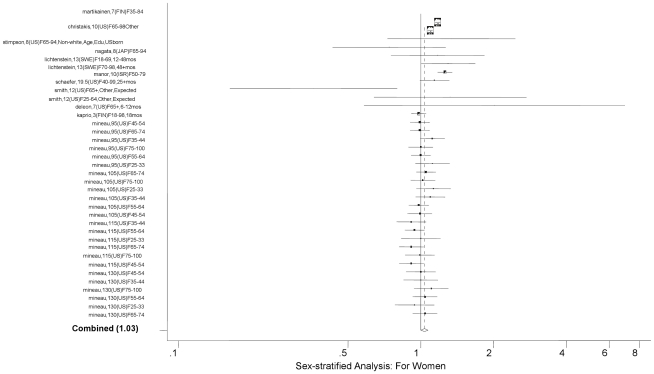
Forest Plot for Women.

**Figure 6:**
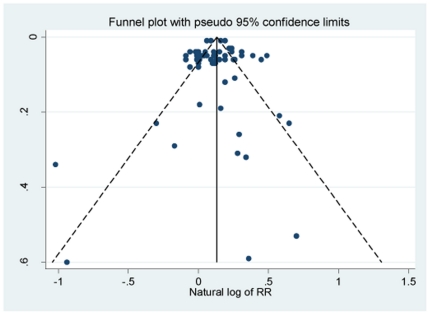
Funnel Plot of Reporting Bias.

**Table 1:** Result of the Primary Meta-Analysis and Stratified Meta-Analyses.

	No. of Studies	No. of Estimates	Overall RR	RR 95% CI	I^2^	I^2^ 95% Uncertainty Interval	p for heterogeneity
Primary analysis	12	69	1.12	1.10, 1.15	88	85, 90	<0.0001
Male-only	11	33	1.22	1.18, 1.26	80	72, 85	<0.0001
Female-only	11	36	1.03	1.00, 1.07	83	77, 87	<0.0001
Age <65 only	6	43	1.18	1.17, 1.25	90	87, 92	<0.0001
Age ≥65 only	9	41	1.10	1.07, 1.13	82	76, 86	<0.0001
Estimates adjusted for more covariates (in addition to age and gender)	12	88	1.14	1.11, 1.18	86	83, 88	<0.0001
Specific models with time since bereavement <6 months	8	20	1.35	1.22, 1.49	87	82, 91	<0.0001
Specific models with time since bereavement ≥6 months	8	19	1.15	1.08, 1.22	76	62, 84	<0.0001

Note: All estimates of overall RRs are based on random-effects models adjusting for between-study heterogeneity.

**Table 2:** Result of the Meta-Regression: Test of Interaction Effect between the Widowhood and Various Study Characteristics.

	Number of studies	RR	95% CI	p-value for difference	Residual heterogeneity (tau^2^)
**Covariate adjustment in addition to age and gender**					
Age- and gender-adjusted only	8	1.12	1.08, 1.17		
Plus other covariates adjusted	4	1.15	0.98, 1.35	0.74	0.015
**Time after bereavement**					
<6 months	10	1.41	1.26, 1.57		
≥6 months	10	1.14	1.10, 1.18	<0.0001	0.016
**Age**					
<65 years of age	6	1.14	1.09, 1.20		
≥65 years of age	6	1.10	1.03, 1.16	0.25	0.015
Gender					
Men	10	1.23	1.18, 1.28		
Women	10	1.04	1.00, 1.08	<0.0001	0.008
**Baseline year**					
Per 50-year increase	12	1.05	1.01, 1.09	0.03	0.014
**Country: US vs. Europe vs. Japan**					
USA	6	1.11	1.07, 1.16	0.62	
Europe	5	1.20	1.07, 1.34	0.75	0.015

## Discussion

In our meta-analysis of cohort studies with 2,263,888 subjects, we found that people experiencing widowhood have an excess risk for premature mortality independent of their age and gender. A unique strength of our analysis and selection criteria was that datasets are mostly nationally representative with sufficient sample sizes. The results, all of which come from longitudinal studies identified in our literature review, seem consistent in showing the widowhood effect, *i.e.*, bereavement is associated with an increased risk of mortality from many causes including suicide. Only limited controls for socioeconomic confounders were available in most studies, and generally even less information was available to adjust for co-morbidities and lifestyle factors. Our results indicate that the effect of widowhood was not significant in studies that controlled for more covariates than age and gender. Although this was based on a low number of included studies that control for other covariates, the result indicates that caution is still needed when discussing whether there is an association of widowhood with mortality or not.

The overall cohort relative risk should also be interpreted with caution, given the substantial heterogeneity detected between studies. Several local factors may be taken into account for this heterogeneity, including the spatial unit across which the widowhood effect was evaluated. Although not evaluated in this study, other contextual characteristics such as national and local social security policies and neighborhood/residential area factors could also explain the heterogeneity between studies. Given the existence of substantial heterogeneity, our primary purpose of this meta-analysis was to formally and quantitatively test the hypotheses on the source of the heterogeneity rather than to get the overall estimates of the effect size. Our analysis by meta-regressions shows quite reasonable results.

Of the significant moderators of the widowhood effect in our results, the most notable one was the time since bereavement. The effect immediately following the spousal loss seems to be more detrimental (<6 months) compared to the longer-term effect, which is consistent in all of the individual studies. The gender difference is also consistently significant in our analysis as well as the individual studies. The difference in baseline year, though fairly small, suggests modest cohort effect.

There are several potential pathways via which marriage, as opposed to widowhood, may protect health [31], [32]. First of all, marriage may increase dispensable income through the principles of economies of scale and specialization of spouses to market and family work, which in turn leads to better health insofar as access to material resources [33]. Second, marriage could provide additional social and emotional support, which may act as a buffer against harmful effects of various psychosocial stress [34]. The guardian role theory suggests that spouses monitor each other, encouraging healthy behaviors of the partner, such as compliance with medical regimens, as well as discouraging unhealthy and risky ones, such as alcohol and other substance use and smoking [31], [35]. Married people, particularly males, have been shown to die less often because of accidents, suicide, homicide, cirrhosis of the liver and diabetes compared to the unmarried [36]. A final hypothesis is that grief associated with the loss of a loved one increases mortality risk directly via psychoneuroimmune pathways. This is especially plausible given that widowhood effect is markedly stronger in the months immediately following the spouse's death than in the years following. Behavioral pathways, and pathways mediated by material resources are likely to influence health in a long-term, cumulative fashion. However, the long-term excess risk associated with widowhood appears quite modest, conditional on surviving the immediate bereavement period.

The following limitations need to be considered while interpreting our findings. First and foremost, all meta-analyses of observational studies are inherently vulnerable to the biases in the original studies [37]. For example, although we evaluated multiple models using alternative sets of covariates, the estimates from the original studies might have been biased by residual confounding. Most of the studies done so far do not seem to have robust socioeconomic controls (i.e., income, wealth, education) at the individual level, all of which are likely to influence mortality and could confound the relationship between widowhood and mortality. Further, there has been very little control of co-morbidities in the existing studies, which exacerbates the bias from inadequate socioeconomic controls. There is a need to learn more about co-determinants of the differential outcomes of bereavement, to understand how the circumstances of bereavement may interact with pre-bereavement experiences, personal socioeconomic and health factors, and ways of coping with grief to cause difficulties. Second, a significant number of studies was excluded because they did not report the necessary information to permit us to include them in the meta-analysis. The omission of such studies might have influenced our conclusions. Third, some of the papers we used only provided age- and gender-stratified analyses but no further control by any other variable, which may have potentially contributed to the high degree of heterogeneity. However, we do not think it seriously compromises the credibility of our findings; rather, our analytical strategy was typical of published meta-analyses. A random-effects meta-analysis addresses the heterogeneity even due to the differences in population (*i.e.*, age groups and gender), under certain assumptions. In general, combining stratified effect estimates is an appropriate way to handle confounding by the stratification variable, assuming no treatment effect heterogeneity, with the only disadvantage that age is a continuous covariate and the stratified models usually categorize age into only a few groups. However, many of the papers we used did not provide a single age-adjusted RR. Fourth, none of the studies done included information on marriage quality or the length of the marriage prior to the study period. Both are potential confounders or modifiers of the observed relationship between individual widowhood and mortality.

Widowhood is a common experience that represents not only a source of personal grief but also appears to predict elevated mortality risk in men and women in diverse populations. The overall association between widowhood and mortality is now well established. The next generation of studies should move beyond this finding and focus on identifying opportunities to improve the health consequences of widowhood. Towards this goal, we identify four important gaps in research on widowhood and mortality. First, further exploration needs to be made on potential mediation and/or confounding. Most prior studies have inadequately controlled for socioeconomic confounders, especially childhood and lifecourse socioeconomic conditions in addition to income, wealth and education, thus leading to uncertainty about whether the widowhood effect is causal. Second, related, the fundamental issue around more meticulous measure of confounders is likely the original study design, rather than the selection of (or lack thereof) covariates at the time of analysis. Cohort studies that have only one-time measurement of exposures, for example measuring exposures at baseline only, cannot handle the confounding and/or mediation issues. More sophisticated longitudinal studies need to be conducted that follow married individuals over time with measurements at many regular intervals, before and after the widowhood event. Third, very few studies have examined physiologic changes underlying the widowhood effect; such analyses could both help improve the causal basis for interpreting widowhood-mortality association, and identify points of intervention. Fourth, despite heterogeneity in widowhood effect sizes, as revealed in prior research, surprisingly little attention has been paid to identifying modifying factors at the individual, familial or contextual level. Identifying modifiers is crucial to understand how to reduce the adverse health consequences of bereavement and as a marker for especially at-risk groups. It is known that the widowhood effect is highest immediately after spousal loss and declines thereafter. Studies that average the effects across multiple time periods obscure the nature of the relationship between widowhood and mortality. Because most widowed individuals at any given moment in time lost their spouse one or more years earlier, studies contrasting currently widowed to currently married will generally produce downwardly biased estimates of the widowhood effect compared to models based on “incident” widowhood. The selection of models is often driven by the available data. In many data sets, the data structure is not appropriate to follow individuals longitudinally from marriage to widowhood.

In conclusion, in a large meta-analysis of prospective studies we observed a robust association between widowhood and mortality. An examination of how far social exposures such as widowhood may vary across individuals and across different contextual factors (including time and place) is critical for improving our understanding of the specific pathways, through which social exposures influence health.

## Supporting Information

Table S1 **PubMed Search Strategy.**
(DOCX)

Table S2 **List of extracted studies.**
(DOCX)

Table S3 **Summary of Selected Studies on Mortality of Bereavement.**
(DOCX)
