# Supplementary material for: Widowhood and Mortality: A Meta-Analysis
Source: PLoS One. 2011 Aug 17;6(8):e23465. doi: 10.1371/journal.pone.0023465 (PMC3157386; doi:10.1371/journal.pone.0023465)
Supplement: Table S1 — PubMed Search Strategy. (DOCX) [file pone.0023465.s001.docx]

**Table S1: PubMed Search Strategy**

**PubMed**

MeSH:

1. Mortality

**Mortality** = All deaths reported in a given population.

Year introduced: 1963

narrower terms include:

**Survival Rate** = The proportion of survivors in a group, e.g., of patients, studied and followed over a period, or the proportion of persons in a specified group alive at the beginning of a time interval who survive to the end of the interval. It is often studied using life table methods.

Year introduced: 1990

**mortality [Subheading]** = Used with human and veterinary diseases for mortality statistics.

2. Widowhood / Bereavement

**Widowhood** = The state of having lost a marital partner by death.

Year introduced: **1994 (includes articles from the 1980s**)

Entry terms:

widows, widow, widowed, widowers, widower

**Bereavement** = Refers to the whole process of grieving and mourning and is associated with a deep sense of loss and sadness.

Year introduced: 1988

narrower term:

**Grief** = Normal, appropriate sorrowful response to an immediate cause. It is self-limiting and gradually subsides within a reasonable time.

Year introduced: 1968

**Spouses** = Married persons, i.e., husbands and wives, or partners. Domestic partners, or spousal equivalents, are two adults who have chosen to share their lives in an intimate and committed relationship, reside together, and share a mutual obligation of support for the basic necessities of life.

Year introduced: 1995 (back indexed to 1970s)

MeSH search:

**("Mortality"[Mesh] OR "mortality "[Subheading]) AND ("Widowhood"[Mesh] OR (("Bereavement"[Mesh] OR "Grief"[Mesh]) AND "spouses"[MeSH Terms]))**

- 71 results

("Mortality"[Mesh] OR "mortality "[Subheading]) AND ("Widowhood"[Mesh] OR widow[tw] OR widows[tw] OR widowed[tw] OR widowhood[tw]) OR ("Bereavement"[Mesh] OR bereavement[tw]) AND (Spouses[Mesh] OR spouse[tw] OR spouses[tw]))

- 495 results

MeSH search with text words for MeSH that don’t go back to 1963; therefore limit search to publications from 1963-1988.

**("Mortality"[Mesh] OR "mortality "[Subheading]) AND ("Widowhood"[Mesh] OR widow[tw] OR widows[tw] OR widowed[tw] OR widowhood[tw]) OR ("Bereavement"[Mesh] OR "Grief"[Mesh] OR bereavement[tw]) AND ("spouses"[MeSH Terms] OR spouse[tw] OR spouses[tw])) AND ("1963"[PDAT] : "1988"[PDAT])**

- 81 results

text words: [tw]

(mortality[tw] OR survival[tw] OR longevity[tw]) AND

(widow[tw] OR widows[tw] OR widowed[tw] OR widowhood[tw])

2 parts:

OR (bereavement[tw] OR grief[tw] OR grieving[tw] OR mourning[tw] OR mourn[tw] OR loss[tw] OR death) AND (spouse[tw] OR spousal[tw])

More terms: fatality

full text word search:

(mortality[tw] OR survival[tw] OR longevity[tw]) AND ((widow[tw] OR widows[tw] OR widowed[tw] OR widowhood[tw]) OR ((bereavement[tw] OR grief[tw] OR grieving[tw] OR mourning[tw] OR mourn[tw] OR loss[tw] OR death[tw]) AND (spouse[tw] OR spousal[tw]))

- 1379 results

Non-indexed articles:

**(mortality[tw] OR survival[tw] OR longevity[tw]) AND ((widow[tw] OR widows[tw] OR widowed[tw] OR widowhood[tw]) OR ((bereavement[tw] OR grief[tw] OR grieving[tw] OR mourning[tw] OR mourn[tw] OR loss[tw] OR death[tw] OR fatality[tw]) AND (spouse[tw] OR spousal[tw])) NOT medline[sb]**

- 44 results

Combined search:

((mortality[tw] OR survival[tw] OR longevity[tw]) AND (widow[tw] OR widows[tw] OR widowed[tw] OR widowhood[tw]) OR ((bereavement[tw] OR grief[tw] OR grieving[tw] OR mourning[tw] OR mourn[tw] OR loss[tw] OR death[tw] OR fatality[tw]) AND (spouse[tw] OR spousal[tw])) NOT medline[sb]) OR (("Mortality"[Mesh] OR "mortality "[Subheading]) AND ("Widowhood"[Mesh] OR widow[tw] OR widows[tw] OR widowed[tw] OR widowhood[tw]) OR ("Bereavement"[Mesh] OR bereavement[tw]) AND ("spouses"[MeSH Terms] OR spouse[tw] OR spouses[tw]) AND ("1963"[PDAT] : "1988"[PDAT])) OR (("Mortality"[Mesh] OR "mortality "[Subheading]) AND ("Widowhood"[Mesh] OR ("Bereavement"[Mesh] AND "spouses"[MeSH Terms])))

- 197 results

((mortality[tw] OR survival[tw] OR longevity[tw]) AND (widow[tw] OR widows[tw] OR widowed[tw] OR widowhood[tw]) OR ((bereavement[tw] OR grief[tw] OR grieving[tw] OR mourning[tw] OR mourn[tw] OR death[tw]) AND (spouse[tw] OR spousal[tw])) NOT medline[sb]) OR (("Mortality"[Mesh] OR "mortality "[Subheading]) AND ("Widowhood"[Mesh] OR widow[tw] OR widows[tw] OR widowed[tw] OR widowhood[tw]) OR ("Bereavement"[Mesh] OR bereavement[tw]) AND ("spouses"[MeSH Terms] OR spouse[tw] OR spouses[tw]) AND ("1963"[PDAT] : "1988"[PDAT])) OR (("Mortality"[Mesh] OR "mortality "[Subheading]) AND ("Widowhood"[Mesh] OR ("Bereavement"[Mesh] AND "spouses"[MeSH Terms])))

- 185 results

**PsycINFO**

There are two search strategies, a broader one that includes both ”Mortality Rate” and “Death and Dying” (used for concepts of dying, mortality), and one that only includes “mortality rate.”

**(DE "Mortality Rate" or DE "Death and Dying") and ((DE "Widows" ) or (DE "Bereavement" OR DE "Grief") and (DE "Spouses" or DE "Human Males"))**

**-** 379 results

This shouldn’t be “human males,” it should be “husbands”, but this yields no results so just remove that part

**(DE "Mortality Rate" or DE "Death and Dying") and ((DE "Widows" ) or ((DE "Bereavement" OR DE "Grief") and DE "Spouses"))**

**- 356 results**

removing “death and dying” severely decreases results:

**(DE "Mortality Rate") and ((DE "Widows" ) or ((DE "Bereavement" OR DE "Grief") and DE "Spouses"))**

- 22 results

Since most of these subject terms were introduced in 1973, I also tried searching using just text words, as we did in PubMed:

(mortality or survival or longevity) and ((widow or widows or widowed or widowhood) or (bereavement or grief or grieving or mourning or mourn or loss or death or fatality) and (spouse or spousal))

To limit this search to 1960-1973, click on revise search, enter the publication years, and click on search again. This retrieves 7 results.
