# Supplementary material for: Widowhood and Mortality: A Meta-Analysis
Source: PLoS One. 2011 Aug 17;6(8):e23465. doi: 10.1371/journal.pone.0023465 (PMC3157386; doi:10.1371/journal.pone.0023465)
Supplement: Table S2 — List of extracted studies. (DOCX) [file pone.0023465.s002.docx]

**Table S2: List of Extracted Studies**

**Extracted:**

1. Christakis NA, Allison PD. Mortality after the hospitalization of a spouse. N Engl J Med. 2006 Feb 16;354(7):719-30.

Elwert F, Christakis NA. The effect of widowhood on mortality by the causes of death of both spouses. Am J Public Health. 2008 Nov;98(11):2092-8. Epub 2008 May 29.

1. Hart CL, Hole DJ, Lawlor DA, Smith GD, Lever TF. Effect of conjugal bereavement on mortality of the bereaved spouse in participants of the Renfrew/Paisley Study. J Epidemiol Community Health. 2007 May;61(5):455-60.
2. Kaprio J, Koskenvuo M, Rita H. Mortality after bereavement: a prospective

study of 95,647 widowed persons. Am J Public Health. 1987 Mar;77(3):283-7.

1. Lichtenstein P, Gatz M, Berg S. A twin study of mortality after spousal bereavement. Psychol Med. 1998 May;28(3):635-43.
2. Manor O, Eisenbach Z. Mortality after spousal loss: are there socio-demographic differences? Soc Sci Med. 2003 Jan;56(2):405-13.
3. a. Martikainen P, Valkonen T. Mortality after death of spouse in relation to duration of bereavement in Finland. J Epidemiol Community Health. 1996 Jun;50(3):264-8.

b. Martikainen P, Valkonen T. Mortality after the death of a spouse: rates and causes of death in a large Finnish cohort. Am J Public Health. 1996 Aug;86(8):1087-93.

c. Martikainen P, Valkonen T. Do education and income buffer the effects of death of spouse on mortality? Epidemiology. 1998 Sep;9(5):530-4.

1. Mendes de Leon C, Kasl S V, Jacobs S. Widowhood and mortality risk in a community sample of the elderly: a prospective study. J Clin Epidemiol 1993; 46: 519–27.
2. Mineau GP, Smith KR, Bean LL. Historical trends of survival among widows and widowers. Soc Sci Med. 2002 Jan;54(2):245-54.
3. Nagata, C., Takatsuka, N., & Shimizu, H. (2003). The impact of changes in marital status on the mortality of elderly Japanese. Annals of Epidemiology, 13, 218–222.
4. Schaefer C, Quesenberry CP, Wi S. Mortality following conjugal bereavement and the effects of a shared environment. Am J Epidemiol 1995; 141: 1142–52.
5. Smith KR, Zick CD. Risk of mortality following widowhood: age and sex differences by mode of death. Soc Biol. 1996 Spring-Summer;43(1-2):59-71.
6. Stimpson JP, Kuo YF, Ray LA, Raji MA, Peek MK. Risk of mortality related to widowhood in older Mexican Americans. Ann Epidemiol. 2007 Apr;17(4):313-9. Epub 2007 Feb 15.

**Not Usable:**

1. Bowling A. Mortality after bereavement: An analysis of mortality rates and associations with mortality 13 years after bereavement. International Journal of Geriatric Psychiatry [serial online]. June 1994;9(6):445-459. *(not enough information)*
2. Cox, P. R., and Ford, J. R. (1964). The Mortality of Widows Shortly After Widowhood. Lancet, 1, 163-164. *(not enough information)*
3. Dupre ME, Beck AN and Meadows SO. 2009. Marital Trajectories and Mortality Among US Adults. American Journal of Epidemiology, 170(5):546-555. *(widowhood measured as a time-invariant, retrospective measures)*
4. Helsing KJ, Comstock GW, Szklo M. Causes of death in a widowed population. Am J Epidemiol. 1982 Sep;116(3):524-32. *(not enough information)*
5. Jacobs S, Kasl S, Ostfeld A, Berkman L, Charpentier P. The measurement of grief: age and sex variation. Br J Med Psychol. 1986 Dec;59 (Pt 4):305-10. *(not enough information)*
6. Johnson NJ, Backlund E, Sorlie PD, Loveless CA. Marital status and mortality: the national longitudinal mortality study. Ann Epidemiol. 2000 May;10(4):224-38. *(not enough information)*
7. Jones DR, Goldblatt PO. Cause of death in widow(er)s and spouses. J Biosoc Sci. 1987 Jan;19(1):107-21. *(not enough information)*
8. Krause AS, Lilienfeld AM (1959). Some epidemiologic aspects of the high mortality rate in the young widowed group. J Chronic Dis 10:207-217 *(not enough information)*
9. Lillard LA, Waite LJ. ‘Til death do us part—marital disruption and mortality. Am J Sociol 1995; 100: 1131–56. *(not enough information, plus widowhood compared to “never married”)*
10. Mellström D, Nilsson A, Odén A, Rundgren A, Svanborg A. Mortality among the widowed in Sweden. Scand J Soc Med. 1982;10(2):33-41. *(not enough information)*
11. Parkes, C. M., Benjamin, B., & Fitzgerald, R. G. (1969). Broken heart: a statistical study of increased mortality among widowers. British Medical Journal, 1(5646), 740-743. *(not enough information)*
12. Pizzetti P, Manfredini M. 'The shock of widowhood'? Evidence from an Italian population (Parma, 1989-2000). Social Indicators Research [serial online]. February 2008; 85(3):499-513.  *(not eligible – not following changes in mortality at onset of bereavement)*
13. Subramanian SV, Elwert F, Christakis N. Widowhood and mortality among the elderly: the modifying role of neighborhood concentration of widowed individuals. Soc Sci Med. 2008 Feb;66(4):873-84. *(population & data overlap with Christakis papers)*
14. Young, M., Benjamin, B., and Wallis, C. (1963). The Mortality of Widowers. Lancet, 2, 454-457. *(not enough information)*
