# Supplementary material for: Widowhood and Mortality: A Meta-Analysis
Source: PLoS One. 2011 Aug 17;6(8):e23465. doi: 10.1371/journal.pone.0023465 (PMC3157386; doi:10.1371/journal.pone.0023465)
Supplement: Table S3 — Summary of Selected Studies on Mortality of Bereavement. (DOCX) [file pone.0023465.s003.docx]

**Table S3: Summary of Selected Studies on Mortality of Bereavement**

|  | **Author Names** | **Dataset Used / Loc.** | **Year(s) of Dataset** | **Sample Size** | **Subject Age Range** | **Primary Cause of Subject Death** | **Findings** | **SES (and/or Demographic) Control** | **Co-morbidities Control** |
| --- | --- | --- | --- | --- | --- | --- | --- | --- | --- |
| *1* | Hart, Hole, Lawlor, Smith & Lever (2007) [[28](#_ENREF_28)] | Renfrew & Paisley /Scotland | 1974 – 1976 (3 yrs follow-up) | 4,395 married couples (8,790) | 45–64 years | All cause (CVD, CHD, Cancer) | Bereaved were at higher risk than non-bereaved (RR=1.27). | Social class, deprivation category | SBP, cholesterol, BMI, height, FEV1, smoking, presence of angina, previous MI. |
| *2* | Kaprio, Koskenvuo & Rita (1987) [[26](#_ENREF_26)] | Finnish Population Register / Finland | 1972 – 1976 (5 yrs follow-up) | 95,647 persons | ≥ 16 years | All Cause; Natural Cause; Violent Cause | Overall RR=1.07. Mortality in the 1^st^ week was > 2-fold the expected rates. | None | None |
| *3* | Christakis & Allison (2006) [[21](#_ENREF_21)] | Medicare / USA | 1993 – 2002 (9 yrs follow-up) | spouses whose partners spent time in hospital before death  widow=252,557, widower=156,004  (Tot 518,240) | ≥ 65 years | All-cause (not specified) | HR for widowers=1.21, widows=1.17 compared with people with a living spouse. Very high increased risk in 1^st^ 30 days post-bereavement | Poverty status (in addition to age, sex and race) – dichotomous, above or below the federal poverty line | None. |
| *4* | Elwert & Christakis (2006) ^[^[^38^](#_ENREF_38)^]^ | Medicare (MedPAR & Vital Status) & SSA / USA | 1993 – 2002 (9 yrs follow-up) | 410,272 couples (4,414 interracial); 86,323 widowers, 176,671 widows | ≥ 67 years | All-cause | Excess mortality in whites (HR 1.55-1.64) but not blacks. Similar in both sexes, especially in the 1^st^ mo, | Poverty status; residential context (crime rate, AMI, unemployment, education) | Charlson score |
| *5* | Lichtenstein, et al (1998) [[12](#_ENREF_12)] | Swedish Twin Registry / Sweden | 1981 – 1993 (12 yrs follow-up) | Spouses; 1,993 pairs of twins, one widows and the other still married, and on 35,860 married individuals. | 64.88 ± 6. 66 yrs (men), 66.25±7.33 yrs (women) | All-cause | Risk higher for the <70 yrs and for the recently widowed (1.78 vs. 1.21). Decrease in risk after 4 yrs in widows <70 yrs | Education (dichotomous, elementary education or high school+) | Smoking status, excessive alcohol drinking, BMI, cardiovascular disease, respiratory disease, other chronic disease |
| *6* | Manor & Eisenback (2003) [[30](#_ENREF_30)] | Israel Longitudinal Mortality Study / Israel | 1983 – 1992 (9.5 yrs follow-up) | 4,420 bereaved men, 11,115 bereaved women; 49,566 men, 41,264 women total | 50-79 years | All-cause | Overall RR=1.25 for men and 1.26 for women. Excess mortality for both bereaved men (1.38) and women (1.48), especially during 1^st^ 6 months | Education (dichotomous), household size, no. of children | None |
| *7* | Martikainen & Valkonen (1996 & 1998) [[9](#_ENREF_9),[25](#_ENREF_25),[27](#_ENREF_27)] | 1985 Finnish Census / Finland | 1986 – 1991 (5 yrs follow-up) | Spouses,  22,294 men, 61,686 women, from 1,580,000 married at start of follow-up | 35-84 years | All-cause, circulatory, accident /violence | Excess mortality 21% (RR=1.21) in men, 9% (rr=1.09) in women. Higher risk for shorter than longer durations of bereavement. Relative mortality similar in different SES groups; absolute difference larger in the lower SES. | Housing tenure, family disposable income, region of residence, language group, size of the household. (3^rd^ paper: education (dich.), personal taxable net income) | None |
| *8* | De Leon, et al (1993) [[13](#_ENREF_13)] | Yale Health & Aging Project (YHAP) / USA | 1982 – 1988 (6 yrs follow-up) | Spouses; 237 widowed from a cohort of 1,046 married (318 spouse pairs among them) | ≥ 65 years | All-cause | 75-100% increased risk in 1^st^ 6 months in elderly widowers, but low power. Higher risk in widows than widowers (65-74 yrs) | Education (yrs), age, race | Smoking, BMI, functional disability status, major chronic conditions |
| *9* | Schaefer, et al (1995) [[7](#_ENREF_7)] | Kaiser Foundation Health Plan (KFHP) / USA | 1973 – 1987 (13 yrs follow-up) | Spouses; 1,453 men, 3,294 women bereaved in cohort of 12,522 spouse pairs | ≥ 40 years | All-cause | Raised RR in both sexes, particularly during 2^nd^ half of first year (RR~2.0) | Age (yrs), race, education (yrs), first marriage or not | Health status, low morale, smoking, alcohol |
| *10* | Smith & Zick (1996) [[22](#_ENREF_22)] | Panel Study of Income Dynamics (PSID) / USA | 1971 – 1982 (12 yrs follow-up) | Spouses  141 men, 351 women, from 1,782 married controls | ≥ 25 years | All-cause | Widowers <65: mortality raised when wife died (at ≤ 6 months); Widows >65: rates lower when husband died of LT illness | None | None |
| *11* | Mineau, Smith & Bean (2002) [[23](#_ENREF_23)] | Utah Population Database / USA | 1895 – 1989 (95 yrs follow-up) | 31,168 couples | ≥ 35 years | All-cause | Significant differences between sexes (excess risk for men) for all ages. | Remarriage (proxy of social support), religion, no. of children born | None |
| *12* | Nagata, Takatsuka & Shimizu (2003) [[29](#_ENREF_29)] | Takayama Study / National  Vital Statistics / Japan | 1992 – 1999 (8 yrs follow-up) | 2,039 male and 1,466 female | 65 – 94 years | All-cause | No evidence for increased mortality (RR=1.01 for mean, 0.74 for women). Instead, a decreased mortality rate among LT widowed women. | Yrs of education, occupation, | BMI, Smoking, Alcohol intake, |
| *13* | Stimpson, Kuo, Ray, Raji & Peek (2007) [[24](#_ENREF_24)] | HEPESE / USA | 1993 – 2000 (8 yrs of follow-up) | 1,693 | ≥ 65 years | All-cause | Widowers higher risk (HR=1.92); no significant effect for widows (1.33). | age, education, US nativity, financial strain, social support | Health behaviors, medical conditions, disability & depressive symptoms |
